# Viral load kinetics and the clinical consequences of cytomegalovirus in kidney transplantation

**DOI:** 10.3389/fimmu.2023.1302627

**Published:** 2024-01-31

**Authors:** Sabina Dobrer, Karen R. Sherwood, Ishan Hirji, James Lan, John Gill, Nancy Matic, Paul A. Keown

**Affiliations:** ^1^Department of Pathology and Laboratory Medicine, University of British Columbia, Vancouver, BC, Canada; ^2^Global Evidence and Outcomes, Takeda Development Center Americas, Inc., Lexington, MA, United States; ^3^Department of Medicine, University of British Columbia, Vancouver, BC, Canada

**Keywords:** kidney transplant, cytomegalovirus, CMV, viral load kinetics, clinical outcomes

## Abstract

**Background:**

Despite advances in clinical management, cytomegalovirus (CMV) infection remains a serious complication and an important cause of morbidity and mortality following kidney transplantation. Here, we explore the importance of viral load kinetics as predictors of risk and potential guides to therapy to reduce transplant failure in a large longitudinal Genome Canada Transplant Consortium (GCTC) kidney transplant cohort.

**Methods:**

We examined the relationship between CMV infection rates and clinical characteristics, CMV viral load kinetics, and graft and patient outcomes in 2510 sequential kidney transplant recipients in the British Columbia Transplant Program. Transplants were performed between January 1, 2008, and December 31, 2018, were managed according to a standard protocol, and were followed until December 31, 2019, representing over 3.4 million days of care.

**Results:**

Longitudinal CMV testing was performed in 2464 patients, of whom 434 (17.6%) developed a first episode of CMV viremia at a median of 120 (range: 9–3906) days post-transplant. Of these patients, 93 (21.4%) had CMV viremia only and 341 (78.6%) had CMV viremia with clinical complications, of whom 21 (4.8%) had resulting hospitalization. A total of 279 (11.3%) patients died and 177 (7.2%) patients lost their graft during the 12 years of follow-up. Patients with CMV infection were at significantly greater risk of graft loss (p=0.0041) and death (p=0.0056) than those without. Peak viral load ranged from 2.9 to 7.0 (median: 3.5) log_10_ IU/mL, the duration of viremia from 2 to 100 (15) days, and the viral load area under the curve from 9.4 to 579.8 (59.7) log_10_ IU/mL × days. All three parameters were closely inter-related and were significantly increased in patients with more severe clinical disease or with graft loss (p=0.001). Duration of the first CMV viremic episode greater than 15 days or a peak viral load ≥4.0 log_10_ IU/mL offered simple predictors of clinical risk with a 3-fold risk of transplant failure.

**Conclusion:**

Viral load kinetics are closely related to CMV severity and to graft loss following kidney transplantation and provide a simple index of risk which may be valuable in guiding trials and treatment to prevent transplant failure.

## Introduction

Cytomegalovirus (CMV) remains one of the most common and serious viral infections following kidney transplantation and is a major therapeutic challenge with profound clinical and economic implications (1–5). Clinical risk varies according to the prior serological status of the donor and recipient, the immunosuppressive therapy employed, the use of viral prophylaxis, and other factors (3, 6, 7). Specific recommendations for both prevention and treatment of CMV infection have been established (1, 2). Recipients at the highest risk for post-transplant infection generally receive continuous antiviral prophylaxis for 3 to 12 months, and pre-emptive therapy is commenced in other settings on detection of systemic viremia by routine long-term monitoring of viral load (3, 6).

However, these treatment strategies are neither benign nor universally effective. Current antivirals have serious toxicities including myelosuppression, which often requires reduction of immune suppression therapy (8), increasing the risk of hospitalization, breakthrough rejection, and graft loss. Additionally, the infection may become refractory or resistant to therapy, the former often associated with impaired immune competence and the latter with viral mutations reducing sensitivity to treatment. As a consequence of these effects, CMV infection increases the risk of premature failure and death, altering the economic costs and benefit of transplantation.

Despite widespread use of viral load monitoring in the management of CMV, we lack precise data to predict outcomes and to guide the use or modification of personalized therapy in kidney transplantation (6). Early evidence from hematopoietic stem cell transplantation indicates that measurement of viral load kinetics may fulfill this need by predicting the risk of death during the first year (9–12). Similar data from the COVID-19 epidemic support the relationship between viral load kinetics and disease outcomes. While preliminary reports propose that this may also pertain to solid organ transplant (13–15), we currently lack formal proof or diagnostic thresholds.

We here confirm the importance of CMV viral load kinetics in kidney transplantation using a large real-world cohort within the Genome Canada Transplant Consortium (GCTC) that is managed according to uniform diagnostic and treatment guidelines. We show that CMV infection increases the risk of both premature graft loss and patient death, while viral load kinetics provide a simple and practical potential marker to highlight risk and to guide therapy. Our goal is now to test these predictions in a structured validation cohort to generate evidence for physicians, payers, and healthcare decision makers that will support the use of CMV viral load kinetics in the clinical transplantation of kidneys, and potentially of other organs.

## Methods

### Study design and study period

This retrospective, longitudinal cohort study was designed to examine the probabilities, risk factors, treatments, and outcomes of CMV infection following kidney transplantation. Current diagnostic procedures for molecular human leukocyte antigen testing, recipient solid phase antibody screening, and viral monitoring were introduced by the British Columbia (BC) Transplant Program in 2008, and standardized therapeutic strategies have been employed since this time. The period from January 1, 2008 to December 31, 2018 was therefore selected as the enrollment period to ensure standardized use of modern diagnostic and therapeutic practices. Patients were followed until December 31, 2019, to ensure a minimum of 1 year follow-up. The study was reviewed and approved by the Institutional Clinical Research Ethics Boards of the University of British Columbia and Vancouver Coastal Health who waived requirement for individual patient consent.

### Data sources

Clinical, laboratory, therapeutic, and outcomes data are maintained in the BC Provincial Kidney Transplant Registry, a single provincial electronic medical record (EMR) system that serves as the basis for patient review and clinical management decisions. Supplementary information was obtained, as required, from additional data systems including the BC Immunology Laboratory, the Renal Transplant Pathology Program, and other sources.

### Patients and follow-up

All patients included in this study underwent kidney transplantation as part of a single integrated provincial program in Vancouver, BC, and were followed medically for the remainder of their transplant course by the BC Transplant Program clinical network. The study included all patients who received a kidney transplant from a living donor (LD) or deceased donor (DD) during the period of observation. Patients were followed up to December 31, 2019, providing a minimum observation of 1 year and a maximum of 12 years, with a total of over 3.4 million days of continuous medical supervision after kidney transplant. Unless otherwise specified, the index date was the date of kidney transplant. Ambulatory clinic follow-up, hospitalizations, episodes of CMV infection, and adverse events including death or graft loss (defined as requirement for dialysis or re-transplantation) were recorded in the EMR.

### Clinical management

Patient evaluation and selection for LD or DD transplantation, donor organ allocation, and all diagnostic, procedural, and therapeutic initiatives were performed according to provincial treatment guidelines, reviewed annually by the BC Transplant Management Committee. Patients considered at low immunological risk (patients who were receiving a first graft from a normal criteria donor, with a calculated panel reactive antibody [cPRA] <80%, who did not have donor-specific antibodies on solid-phase assay) received basiliximab, tacrolimus, mycophenolate mofetil, and rapid prednisone elimination. Those at higher risk normally received antithymocyte globulin (ATG) for induction therapy, tacrolimus, mycophenolate mofetil, and long-term prednisone treatment. Immune suppression was adjusted uniquely by the transplant team according to the time post-transplant, the clinical status, and therapeutic concentrations of individual drugs according to a standard provincial management protocol. Graft biopsy was performed for cause and reviewed by a central team of kidney transplant pathologists.

### CMV testing

CMV serological status at the time of transplantation was determined for both the donor (D) and recipient (R) according to the presence or absence of CMV IgG antibodies reported on pre-transplant testing. Four CMV risk groups were defined, categorized as D−/R−, D−/R+, D+/R−, and D+/R+.

Measurement of CMV viral load was performed by St. Paul’s Hospital Virology Laboratory using quantitative PCR methodology. CMV viral load testing was performed using the cobas^®^ CMV quantitative nucleic acid test on the cobas^®^ 6800 (Roche Diagnostics) calibrated to the World Health Organization (WHO) CMV standard in IU/mL. Prior to June 2017, CMV viral load testing had been performed using a laboratory-developed test targeting the CMV glycoprotein B (UL55) gene in IU/mL; prior to October 2014, this assay had been reported in copies/mL (16). Recipient testing was performed pre- and post-transplant as part of routine bloodwork taken approximately weekly for the first 4 weeks, then every 2 weeks to Month 3, every 1 to 2 months to Month 12, and as required after the first post-transplant year. CMV testing was intensified for 6 months following prophylaxis or treatment of infection independent of the time in the transplant course. A CMV DNA viral load of ≥1000 copies/mL or ≥830 IU/mL was the threshold for commencement of treatment (17–19). The duration of CMV viremia was defined as the time in days from the first to the last positive CMV test for the episode. To ensure uniform interpretation, the commencement and completion of viremic episodes were confirmed by 2 consecutive tests performed within 30 days. Response was defined as decline in viral titers below the threshold of treatment (≥1000 copies/mL or ≥830 IU/mL).

### CMV prophylaxis and treatment

Based on BC Transplant Program clinical guidelines for kidney transplantation, adult and pediatric patients who were CMV seronegative and who received a graft from a seropositive donor (D+/R−), CMV positive pediatric patients, and patients who received ATG induction therapy were treated with valganciclovir prophylaxis for 3 to 6 months, which normally commenced on the day of, or immediately following, transplant. Patients who developed CMV viremia above the treatment threshold but were not on prophylaxis received pre-emptive valganciclovir treatment for at least 3 weeks until the viremia resolved. Treatment was administered at a dose of 900 mg orally twice daily, or 5 mg/kg intravenously twice daily, adjusted for kidney function and leukopenia. CMV monitoring was performed weekly during the episode of viremia and repeated monthly for at least 2 months after an episode of infection or termination of prophylaxis. Specific medications, doses, and duration of each therapy were obtained from the BC Provincial Transplant database based on pharmacy dispensing data. Treatment episodes were considered separate when the time between courses of therapy was >7 days. Different agents and dosages could be administered within 1 continuous treatment episode.

### CMV related outcomes

CMV-related outcomes were defined based on the guidelines of the American Society of Transplantation Infectious Disease Community of Practice 2019 (1) adjusted to reflect the longitudinal data available in the EMR. Since CMV end-organ disease was difficult to classify in an observational retrospective study given the many potential and concomitant causes for gastrointestinal, hepatic, pulmonary, and other dysfunction, this was included in the CMV clinical syndrome.

CMV infection was considered as an episode of CMV viremia, determined by the presence of a viral titre exceeding the laboratory threshold, with or without other manifestations. The duration of a CMV viremic episode was defined as the time in days from the first to the last positive CMV test. Recurrence of viremia following resolution of a first infection was designated as a second or subsequent infection and was not considered in this study.

CMV viremia alone was considered as the presence of CMV replication with a viral load of ≥1000 copies/mL, based on prior literature (20–22), or ≥830 IU/mL without corresponding laboratory abnormalities or clinical signs and symptoms of disease.

CMV disease was defined as CMV viremia with classical clinical features of CMV infection including at least one of the following laboratory abnormalities: leukopenia or neutropenia, thrombocytopenia or elevated hepatic transaminases (conventionally described as CMV syndrome), and/or organ injury, defined as presence of gastrointestinal disease, pneumonitis, hepatitis, nephritis, myocarditis, pancreatitis, encephalitis, retinitis, or pulmonary or other classical features (described as CMV end-organ disease). CMV syndrome and end-organ disease were combined in this analysis because of the heterogeneity of presentation and difficulty in determining causality of organ involvement, which may be due to other infections, drug toxicity, or other causes not clearly distinguishable in a retrospective observational study. Management was classified as ambulatory or hospitalized depending on whether the patient was managed as an outpatient or an in-patient.

### Statistical methods

#### Data review

Data review was performed using visualization, tabulation, and other requisite computational processes; missing data were noted but were not imputed for this analysis, and all data discrepancies were reviewed and approved by the research team. Patients were included from the day of transplant surgery (Day 0) and censored at death or end of the study period. Patients were stratified according to donor source (DD or LD), the number of previous transplants (0 or 1+), and donor/recipient CMV serological status as required.

#### Descriptive statistics

Continuous variables were summarized using the number of non-missing observations, mean, standard deviation (SD), median, minimum, and maximum values. Categorical variables were summarized using the number and percentage of subjects belonging to each category. The relationships between CMV infection and patient and graft outcomes were determined independently. Pearson’s Chi-squared test (χ²) was used for comparison of categorical variables by different stratifications. For continuous variables, a non-parametric, two-sided Mann–Whitney U test was used for 2 strata (e.g., living/diseased donor, number of previous transplants) and a non-parametric Kruskal–Wallis H test for 3 or more strata categories (e.g., serological donor/patient status). The time of onset of CMV viremia was calculated using conventional descriptive measures for the mean and distribution, and depicted graphically by baseline donor and recipient CMV serological status, by number of CMV viremia episodes, and by clinical presentation using kernel density estimation.

#### Regression modeling

Multivariable statistical models were developed to adjust for the influence of various covariates on principal independent outcomes of CMV disease, and graft and patient survival. These included: baseline recipient variables (primary disease diagnosis, age at transplant, sex, race, number of prior transplants); donor/recipient CMV variables (donor and recipient CMV serological mismatch); donor/transplant variables (type of donor, donor age, sex, race); induction immunosuppression (use of ATG or other biologics); and delayed graft function.

#### Survival analysis

To assess the relationship between long-term consequences of CMV infection including the impact on graft and overall survival, Kaplan–Meier plots with survival probability compared using the logrank test, and cumulative incidence estimates using Gray’s test were used. Unadjusted and adjusted hazard ratios (HR) were calculated with a Cox proportional hazard model. The log(−log[survival]) versus log of survival time and interaction with times for categorical variables and Schoenfeld Residuals for continuous variables were used to check Cox proportionality assumptions.

#### Viral load kinetics

Viral load kinetics were calculated using maximum viral load, duration of viremia in days, and the integral of viral load over time. The integral viral load was calculated for each patient episode using the trapezoidal method to produce an area under the curve (AUC). An adjusted receiver operator characteristic (ROC) curve was used to show the diagnostic probability of CMV viral load kinetics in predicting patient death or graft failure. The maximum Youden statistic was used to evaluate sensitivity and specificity estimates.

## Results

### Patient cohort

**Figure 1:**
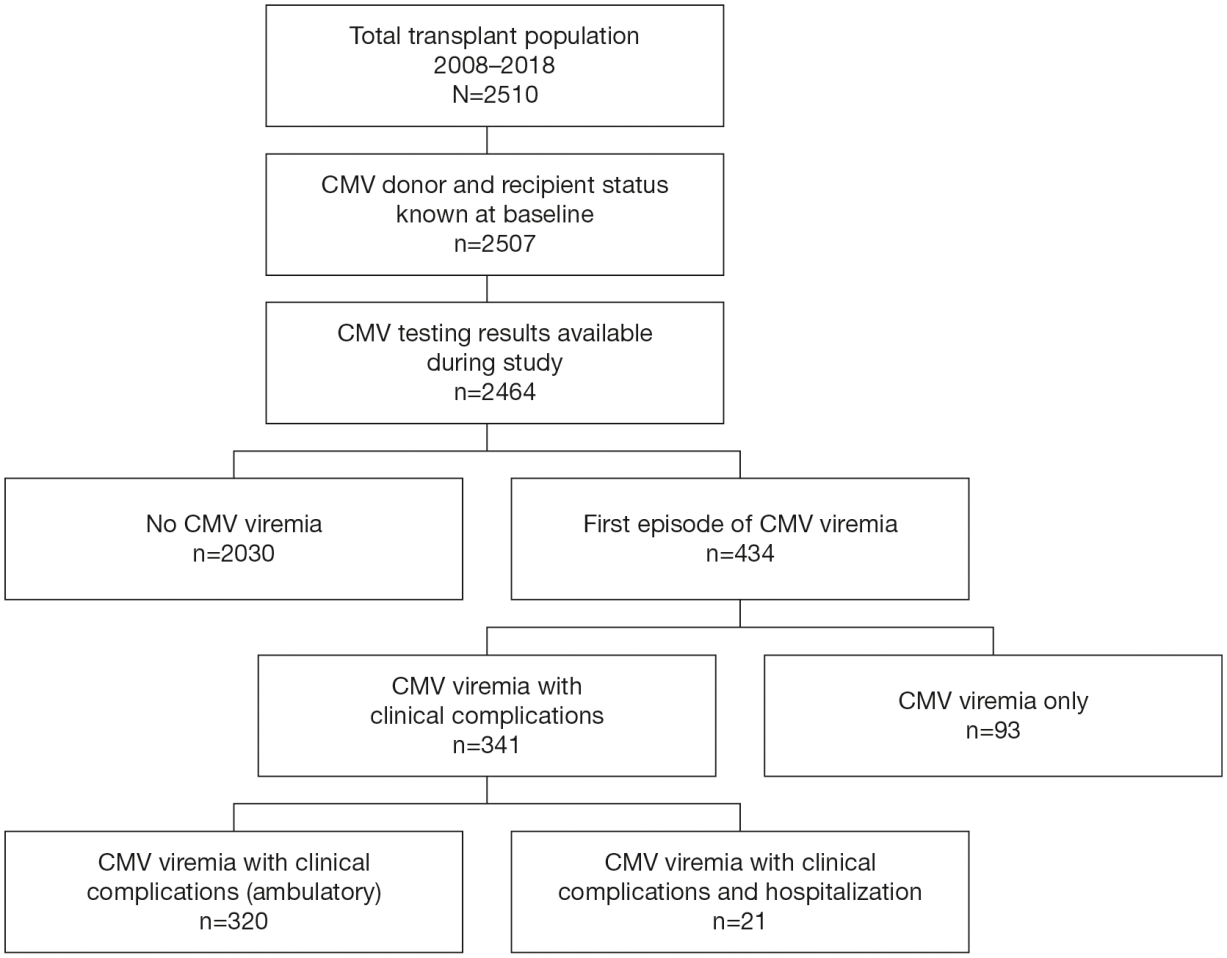
Patient Disposition. Disposition of patients in the study cohort who received a kidney transplant within the observation period of 2008–2018.

A total of 2510 patients received a kidney transplant during the period of observation; 1441 received a DD graft and 1069 an LD graft; 2324 patients had no prior transplants while 186 (7.4%) had 1 or more prior transplants. Donor and recipient CMV serostatus were available in 2507 (99.9%) patients and post-transplant CMV viral load measurement was recorded in 2464 (98.2%) patients, who form the basis of this analysis (**Figure 1**). As shown in **Table 1**, 62.7% of these recipients were male, the median age was 54.1 (range: 2–82 years) years, and 3.0% were pediatric patients (<19 years). In all, 1568 recipients and 1337 donors were CMV seropositive, and 35.8% of donor/recipient pairs were D+/R+, 18.4% were D+/R−, 27.8% were D−/R+, and 17.9% were D−/R−.

**Table 1:** Patient demographics, primary diagnosis, antiviral prophylaxis, death rate, and graft failure rate by CMV risk group.

	D−/R− (a)	D−/R+ (b)	D+/R− (c)	D+/R+ (d)	Total^†^	p value^‡^
Total	442	685	454	883	2464	
	(100.0)	(100.0)	(100.0)	(100.0)	(100.0)	
Sex						
Female	146 (33.0)	274 (40.0)	149 (32.8)	350 (39.6)	919 (37.3)	0.009
Male	296 (67.0)	411 (60.0)	305 (67.2)	533 (60.4)	1545 (62.7)	
Age <19 years*						
Yes	19 (4.3)	11 (1.6)	26 (5.7)	19 (2.2)	75 (3.0)	0.000
No	423 (95.7)	674 (98.4)	428 (94.3)	864 (97.8)	2389 (97.0)	
Age, years*						
Minimum	2.2	9.0	1.7	6.8	1.7	0.000
Median	51.0	55.3	51.3	56.0	54.1	
Maximum	80.3	81.1	80.0	82.0	82.0	
Standard deviation	16.0	13.8	16.2	14.9	15.2	
Mean	48.8	53.7	49.0	53.5	51.9	
Mean 95% CI	(47.3, 50.3)	(52.6, 54.7)	(47.5, 50.5)	(52.5, 54.5)		
Race						
Caucasian/White	391 (88.5)	326 (47.6)	381 (83.9)	374 (42.4)	1472 (59.7)	0.000
Other	51 (11.5)	359 (52.4)	73 (16.1)	509 (57.6)	992 (40.3)	
Primary diagnosis						
Glomerulonephritis	143 (32.4)	233 (34.0)	153 (33.7)	299 (33.9)	828 (33.6)	0.156
Diabetes	88 (19.9)	165 (24.1)	88 (19.4)	214 (24.2)	555 (22.5)	
Other	211 (47.7)	287 (41.9)	213 (46.9)	370 (41.9)	1081 (43.9)	
Prophylaxis^§^						
Yes	70 (15.8)	289 (42.2)	435 (95.8)	329 (37.3)	1123 (45.6)	
No	372 (84.2)	396 (57.8)	19 (4.2)	554 (62.87)	1341 (54.4)	
Duration of prophylaxis^§^, days						
Minimum	2.0	2.0	3.0	2.0	2.0	0.000
Median	32.5	57.0	180.0	62.0	82.0	
Maximum	867.0	613.0	1638.0	596.0	1683.0	
Standard deviation	106.3	59.0	119.1	52.6	103.4	
Mean	51.0	65.4	174.1	68.9	107.6	
Mean 95% CI	(25.6, 76.3)	(58.6, 72.3)	(162.9, 185.3)	(63.2, 74.6)		
Death						
Yes	47 (10.6)	67 (9.8)	56 (12.3)	109 (12.3)	279 (11.3)	0.360
No	395 (89.4)	618 (90.2)	398 (87.7)	774 (87.7)	2185 (88.7)	
Graft failure						
Yes	37 (8.4)	31 (4.5)	42 (9.3)	67 (7.6)	177 (7.2)	0.010
No	405 (91.6)	654 (95.5)	412 (90.7)	816 (92.4)	2287 (92.8)	

Data are n (%) unless indicated otherwise.

*At first transplant.

^†^2464 patients had at least one CMV viremia test done during the study period.

^‡^p value for comparison by CMV mismatch group.

^§^Prophylaxis refers to any administration of CMV treatment drugs within first 14 days post-transplant.

CI, confidence interval; CMV, cytomegalovirus; D, donor; R, recipient.

### CMV prophylaxis and therapy

Of the 2464 recipients with CMV virological data, 1123 (45.6%) received antiviral prophylaxis, principally with valganciclovir or ganciclovir sodium. Of these, 38.7% were D+/R−, 29.3% were D+/R+, 25.7% were D−/R+, and 6.2% were D−/R−. Prophylaxis continued for a median of 82 (range: 2–1683) days and was longest in D+/R− recipients (180 days) and shortest (32.5 days) in D−/R− recipients. A total of 294 CMV infections occurred in the 1123 patients receiving prophylaxis (26.2%) compared with 223 infections in 1341 patients (16.6%) not receiving prophylaxis.

### CMV viremia

A total of 434 patients (17.6%) developed CMV infection, of whom 345 patients (79.5%) received a kidney from a CMV seropositive and 89 (20.5%) from a CMV seronegative donor. The CMV infection rate was highest in D+/R− graft recipients (155/454, 34%), followed by D+/R+ (190/883, 22%), D−/R+ (85/685, 12%), and D−/R− groups (4/442, 1%). Less than 0.5% of viremia episodes occurred during active antiviral prophylaxis.

**Figure 2:**
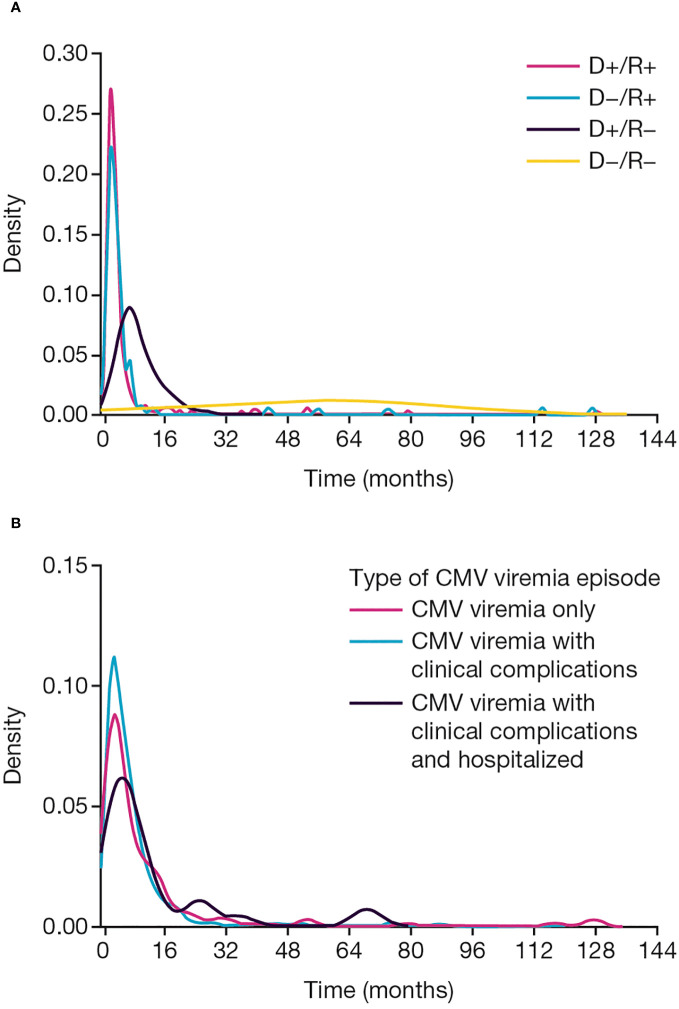
CMV viremia onset. Time to onset of CMV viremia **(A)** by baseline CMV donor and recipient serological status, **(B)** by clinical presentation. CMV, cytomegalovirus; D, donor; R, recipient.

The first episode of infection commenced a median of 120 (range: 9–3906) days post-transplant (**Figure 2**). Time to onset was shortest in D+/R+ recipients (median: 81 days, range: 9–3906 days), and longest in D−/R− recipients (median: 1794 days, range: 365–2328 days; p<0.001).

Of the 434 first episodes of CMV viremia, CMV viremia alone, without laboratory abnormalities or classical clinical features, accounted for 93 (21.4%). CMV disease (combining clinical syndrome and end-organ disease) with clinical or laboratory evidence of systemic disease or target organ injury occurred in 320 (73.7%) of first CMV episodes, and 21 patients (4.8%) were hospitalized for care during a first episode of CMV infection (**Figure 1**).

### Patient and graft outcomes

**Figure 3:**
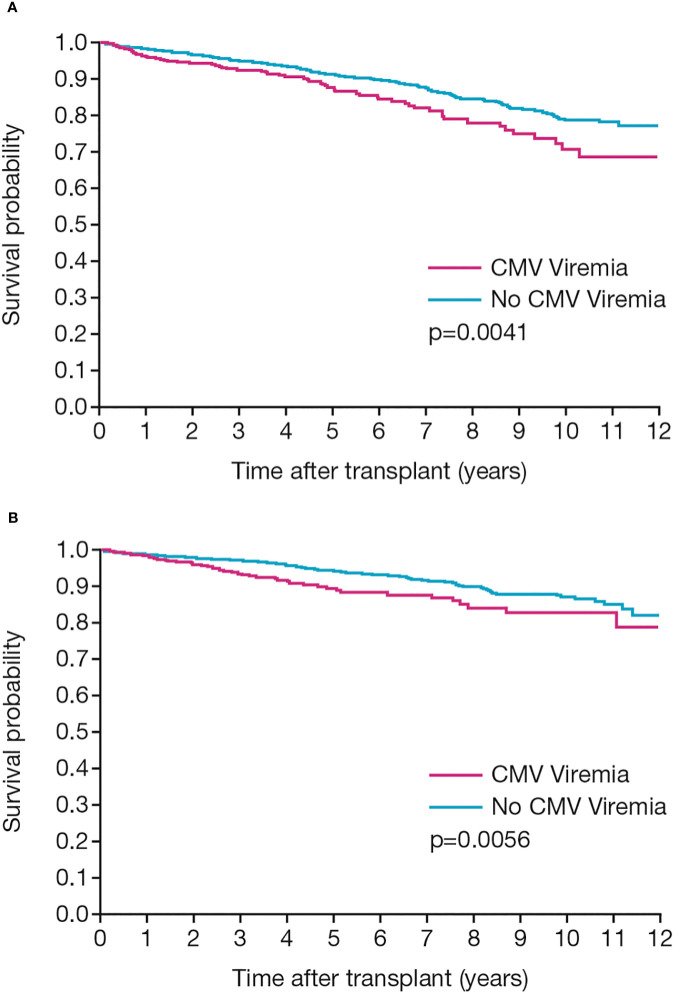
Patient and graft outcomes. Kaplan-Meier analyses show **(A)** patient survival and **(B)** death-censored graft survival in patients with or without CMV infection post-transplant.

A total of 279 patients (11.3%) died and a further 177 patients (7.2%) lost their graft during the period of follow-up. CMV serological status at baseline was not associated with either mortality or graft loss. However, patient and graft survival were significantly related to the occurrence of CMV infection. Both survival measures were significantly reduced in patients who developed CMV infection (p=0.0041 and p=0.0056, respectively; **Figures 3A, B**).

### CMV viral load kinetics

**Figure 4:**
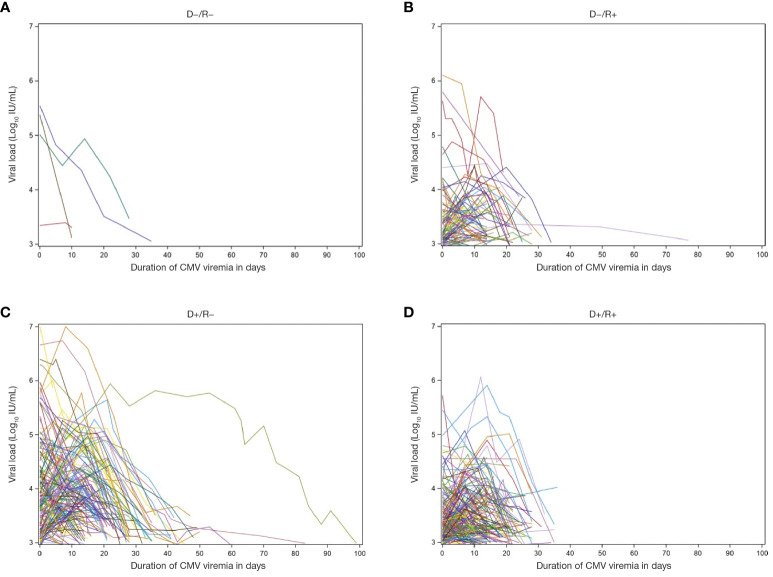
Viral load and duration of viremia. Patient-level depiction of the heterogeneity of both magnitude of viral load and duration of viremia among patients with documented CMV infection post-transplant (n = 434). Figure shows patients with Donor/Recipient status of **(A)** D−/R−, **(B)** D−/R+, **(C)** D+/R−, and **(D)** D+/R+. D, donor; CMV, cytomegalovirus; R, recipient.

Duration of CMV viremia ranged from 2 to 100 days with a median of 15 days. The heterogeneity of viremia is shown in **Figure 4**. Duration was independent of the donor source (LD vs DD) or the number of prior transplants, although 10 of the 11 subjects with duration >40 days were D+/R−. Median duration increased slightly according to clinical presentation, from 15 (range: 5–36) days in patients with viremia only to 16 (range: 2–114) days in those with clinical syndrome and 17 (range: 3–84) days in those admitted to hospital during the episode.

The maximum viral load measured during the first episode of viremia ranged from 2.9 to 7.0 log_10_ IU/mL with a median of 3.5 log_10_ IU/mL. All 5 patients with a maximum viral load >6 log_10_ IU/mL were D+/R−. Median values increased from 3.6 (range: 3.0–5.9) log_10_ IU/mL in patients with CMV viremia alone to 3.9 (range: 2.9–7.0) log_10_ IU/mL in patients with CMV disease and to 4.6 (range: 3.2–6.4) log_10_ IU/mL in those with CMV disease who were admitted to hospital (p<0.0001).The integral (AUC) of viral load over time ranged from 9.4 to 579.8 log_10_ IU/mL × days with a median of 59.7 log_10_ IU/mL × days. Median values increased from 55.6 (range: 16.1–223.7) log_10_IU/mL × days in patients with viremia only to 68.3 (range: 9.5–579.8) log_10_ IU/mL × days in those with clinical syndrome, and 91.3 (range: 9.7–347.8) log_10_ IU/mL × days in those admitted to hospital during the episode.

**Figure 5:**
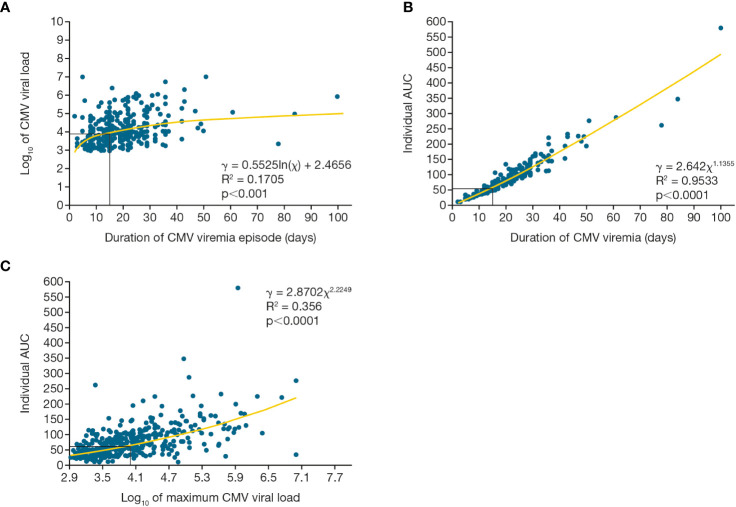
Viral load kinetics over time for first CMV viremia episode. Relationship between maximum CMV viral load, duration of viremia, and integral of the CMV viral load as a function of time (AUC) for the first episode of CMV viremia. Figure **(A)** shows maximum viral load and duration of viremia, **(B)** shows viral load AUC and duration of viremia, **(C)** shows viral load AUC and maximum viral load. AUC, area under the curve; CMV, cytomegalovirus.

The three indices of viral load kinetic measures during the first viremic episode were tightly correlated. **Figure 5** shows the significant relationship between maximum viral load and duration of CMV viremia (p=0.001), between CMV AUC and duration of CMV viremia (p<0.0001), and between peak viral load and the AUC of viremia (p<0.0001).

### Developing robust and pragmatic predictors

**Figure 6:**
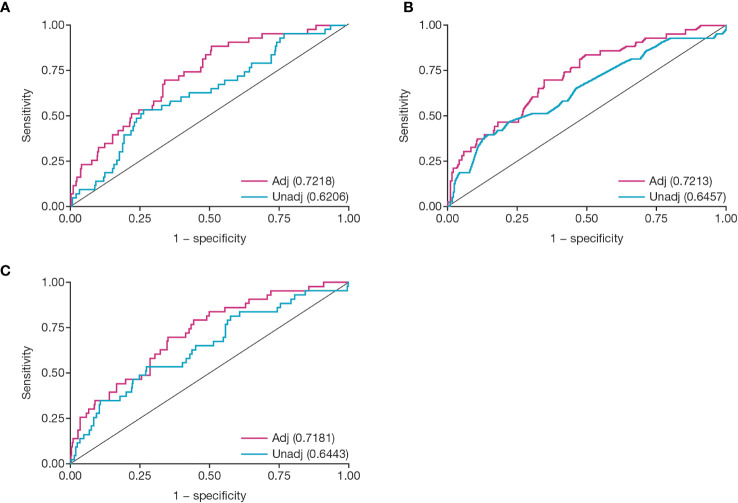
Receiver operator characteristic analysis for CMV viral load kinetics. Figures show ROC analysis of: **(A)** peak viral load, **(B)** duration of viremia in weeks, and **(C)** viral load over time (AUC). Numbers given in parentheses are area under the unadjusted or adjusted ROC curve. Covariates for each adjusted model were delayed graft function, Induction ATG, and CMV D/R status at transplant. Adj, adjusted; ATG, antithymocyte globulin; AUC, area under the curve; CMV, cytomegalovirus; D/R, donor/recipient; ROC, receiver operator characteristic; unadj, unadjusted.

ROC curves were constructed to examine the diagnostic probabilities of transplant failure. Adjusted ROC analysis of CMV peak viral load provided an AUC of 0.72 (**Figure 6A**; 95% confidence interval [CI]: 0.65, 0.80); for duration of viremia, the AUC was 0.72 (**Figure 6B**; 95% CI: 0.64, 0.80); and for viral load over time, the AUC was 0.72 (**Figure 6C**; 95% CI: 0.40, 0.80).

**Figure 7:**
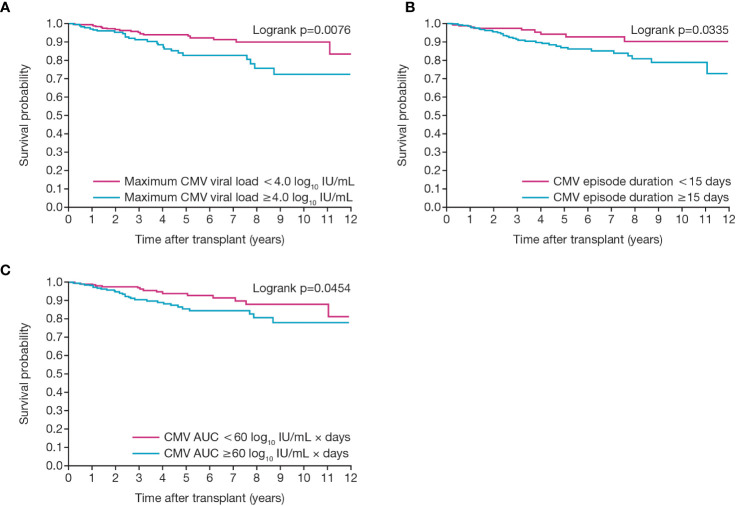
Relationship between death-censored graft survival and CMV viral load kinetics. Figures show graft survival according to first CMV infection in patients with **(A)** maximum CMV viral load of greater or less than 4.0 log_10_ IU/mL and **(B)** duration of viremia of greater or less than 15 days and **(C)** an individual AUC of greater or less than 60. AUC, area under the curve; CMV, cytomegalovirus.

To create a pragmatic set of clinical predictors, we selected 15 days (the median duration of the first episode of CMV viremia) along with the closely corresponding values of peak viral load of 4.0 log_10_ IU/mL and an AUC of 60 log_10_ IU/mL × days (**Figure 7**).

Unadjusted Cox proportional hazard modeling showed that all 3 viral load kinetic parameters were significantly and directly related to the probability of graft failure with HRs above 1 and narrow 95% CIs (**Table 2**). For CMV duration (in weeks), the HR was 1.164 (95% CI: 1.039, 1.304; p=0.0088); for CMV viral load (log_10_ IU/mL), the HR was 1.505 (95% CI: 1.069, 2.117; p=0.0190); and for individual AUC 100 (log_10_ IU/mL) × days), the HR was 1.475 (95% CI: 1.096, 1.987; p=0.0104).

**Table 2:** Cox proportional hazard modeling of the effect of viral load kinetics on graft survival adjusted for other risk factors.

	Unadjusted models		Adjusted AUC model		Adjusted viral load model		Adjusted duration model	
	HR (95% CI)	p value	HR (95% CI)	p value	HR (95% CI)	p value	HR (95% CI)	p value
Individual AUC	1.475 (1.096, 1.987)	0.0104	1.503 (1.052, 2.147)	0.0252				
CMV viral load	1.505 (1.069, 2.117)	0.0190			1.557 (1.049, 2.311)	0.0281		
Duration of CMV episode	1.164 (1.039, 1.304)	0.0088					1.170 (1.022, 1.339)	0.0225
CMV D/R status at transplant								
D−/R−	N/A	N/A	N/A	N/A	N/A	N/A	N/A	N/A
D−/R+	0.203 (0.048, 0.862)	0.0307	0.161 (0.038, 0.686)	0.0135	0.162 (0.038, 0.689)	0.0137	0.160 (0.038, 0.680)	0.0130
D+/R−	0.970 (0.523, 1.798)	0.9233	1.126 (0.563, 2.254)	0.7365	0.968 (0.458, 2.047)	0.9323	1.131 (0.568, 2.251)	0.7256
D+/R+	Reference							
Induction ATG								
Yes	2.299 (1.253, 4.218)	0.0072	2.217 (1.185, 4.150)	0.0128	2.225 (1.186, 4.176)	0.0127	2.140 (1.141, 4.013)	0.0177
No	Reference							
Delayed graft function								
Yes	2.513 (1.366, 4.624)	0.0031	2.700 (1.422, 5.126)	0.0024	2.663 (1.403, 5.055)	0.0027	2.694 (1.422, 5.105)	0.0024
No	Reference							

AUC units are 100 (log_10_ IU/mL)*days. Viral load units are log_10_ IU/mL. Duration units are weeks.

ATG, antithymocyte globulin; AUC, area under the curve; CI, confidence interval; CMV, cytomegalovirus; D, donor; HR, hazard ratio; N/A, not applicable; R, recipient.

Unadjusted models explored a range of covariates, identifying the three most important in contributing to CMV infection to be the use of ATG induction immunosuppression (unadjusted HR: 2.299 [95% CI: 1.253, 4.218], p=0.0072), delayed graft function (unadjusted HR: 2.513 [95% CI: 1.366, 4.624], p=0.0031), and CMV status at baseline with D+/R− as the reference group (D+/R− unadjusted HR: 0.970 [95% CI: 0.523, 1.798], p=0.9233; D−/R+ unadjusted HR: 0.203 [95% CI: 0.048, 0.862], p=0.0307).

Adjusted multivariable Cox models incorporating these covariates then provided an adjusted hazard ratio for CMV duration of 1.170 (95% CI: 1.022, 1.339; p=0.0225), indicating a 17% increase in risk of graft failure for every additional week of CMV duration. The hazard ratio for viral load of 1.557 (95% CI: 1.049, 2.311; p=0.0281) indicated a 56% increase in risk of graft failure for each log_10_ increase in viral titer, and the hazard ratio for AUC of 1.503 (95% CI: 1.052, 2.147; p=0.0236) indicated a 50% increase in risk of graft failure for each increase of 100 AUC units (log_10_ IU/mL × days) across the observed range of 9 to 580 units.

## Discussion

Accurate monitoring of viral load and rigorous use of antiviral therapy have mitigated the devastating consequences of CMV infection and modulated the important early indirect consequences and costs of care. Introduction of a common reference material, the first WHO International Standard for Human Cytomegalovirus for Nucleic Acid Amplification Techniques (CMV WHO IS; NIBSC code 09/162) and Standard Reference Material (SRM) 2366 Cytomegalovirus for DNA Measurements from the National Institute of Standards and Technology, has enabled laboratories to unify the reporting of CMV viral loads (19).

This study, one of the largest longitudinal studies of “real-world evidence” in current practice to examine the incidence, expression, and consequences of CMV infection post-transplant, confirms that CMV remains a common and serious complication of kidney transplantation despite rigorous application of treatment guidelines with standardized use of antiviral prophylaxis, viral monitoring, and pre-emptive therapy. Overall, 18% of patients transplanted developed a first episode of CMV infection and 3% experienced additional episodes, with most occurring during the first post-transplant year. One quarter of cases presented as asymptomatic viremia, three quarters as CMV disease with hematological, gastrointestinal, hepatic, pulmonary, ocular, and other consequences and 5% were hospitalized with CMV disease (1, 23). Patient and graft survival were significantly reduced in patients who experienced CMV infection (p<0.004–0.006) and were profoundly diminished in those with more than 1 episode of viremia (p<0.001).

Precise predictors are critical to guide therapy and enable early effective treatment in order to mitigate these devastating complications. Viral load surrogate endpoints have revolutionized the management of serious and potentially lethal viral infections including HIV, hepatitis, and COVID-19 and have transformed the clinical evaluation and time to licensure for new agents in these infections. Monitoring of CMV viremia has greatly advanced the management of post-transplant infection, but results are normally considered as quasi-binary around a pre-selected treatment threshold. We show here for the first time that viral load kinetics serve as important predictors of premature transplant failure, and that the more comprehensive data encompassed within the broad dynamic ranges in peak viral load (range: 3–7 log_10_ IU/mL), duration of viremia (range: 2–100 days), and integral of viral load over time (AUC) (range: 9–586 IU/mL × days) serve to identify those at maximum risk. A simple heuristic using peak viral load >10,000 IU/mL, duration of viremia >15 days, or an AUC of >60 log_10_ IU/mL × days may serve as a valuable tool to inform early and active therapy and provide a potential surrogate marker in clinical trial settings.

In concert with other reports, CMV infection was most prevalent (34%), despite prophylaxis, in seronegative recipients of organs from a seropositive donor (D+/R−), was intermediate in CMV seropositive recipients (D+/R+), and low in CMV seronegative recipients of organs from seronegative donors (D−/R−) (1, 24). Guidelines in Canada, the USA, and Europe recommend the use of prophylaxis for high-risk groups based on cost–benefit analyses and risks of leukopenia and other complications. However, within-center guidelines are often more nuanced and treatment individualized based on other risk factors (for example, ATG may increase risk of CMV and mammalian target of rapamycin inhibitors [mTORi] may reduce risk) (25, 26). Consequently, prophylaxis is often used more extensively and for longer duration in many centers in the USA versus Canada and Europe (3, 27), and large-scale analysis of the United States Renal Data System indicates that CMV infection rates are reduced along with other opportunistic infections and post-transplant diabetes in these patients.

There are a number of potential limitations associated with this study, including selection bias, information bias and confounding, which are inherent to observational design. To minimize selection bias, the study included all sequential patients transplanted in a single program in Canada who were followed throughout the period of observation within an integrated care network. While information bias may occur from many sources, stringent efforts were made to reduce this, using a single provincial electronic database with standard entry practices and uniform analytical strategies. Risk strata and classification criteria were defined according to national and international norms, and the period of enrollment and observation was chosen to ensure standardized diagnostic and therapeutic practices. Although confounding is perhaps more difficult to eliminate, we have made stringent efforts to minimize confounding by indication or by patient risk through *post hoc* stratification and regression modeling, and while the potential for time-varying differences in patient referral, case mix, unit services, and care patterns remain, these reflect normal practice patterns over this period.

Despite the limitations inherent in observational design, this large, longitudinal study has important strengths including sample size and provincial scope, the inclusion of sequential patients transplanted within a standardized care program, and long-term follow-up and management within a uniform clinical and laboratory program. It confirms the serious consequences of CMV infection, which not only causes systemic illness but also triggers inflammatory injury of specific target organs, complicates effective immunosuppression, destabilizes host immunological quiescence, and jeopardizes both graft and patient survival. The relationship between CMV infection and transplant failure may be causal, related to direct systemic endothelial injury, to the immune modulating effect of the virus in enhancing targeted T-cell rejection, or to iatrogenic reduction in immune suppression secondary to leukopenia, all leading to progressive vascular destruction, or may be consequential where treatment of rejection increases the risk of viremia (28). We cannot decipher all these interactions at present, which are now the focus of a deeper evaluation. However, the data reported here underscore this adverse consequence of the virus and demonstrate that the simple application of standardized clinical guidelines does not prevent the ravages of this infection. We show here that CMV viral load kinetics are important in predicting outcome and provide a simple pragmatic set of predictor values that may be critical in guiding therapy and may serve as an important virological endpoint for therapeutic trials in this disease.

## Data availability statement

The datasets presented in this article are not publicly available because they are confidential health services data from British Columbia. Requests to access the datasets should be directed to Dr. Paul Keown on behalf of the British Columbia Transplant Organization.

## Ethics statement

The studies involving humans were approved by Institutional Clinical Research Ethics Boards of the University of British Columbia and Vancouver Coastal Health. The studies were conducted in accordance with the local legislation and institutional requirements. The ethics committee/institutional review board waived the requirement of written informed consent for participation from the participants or the participants’ legal guardians/next of kin because the study received an ethical review by the Institutional Clinical Research Ethics Boards of the University of British Columbia and Vancouver Coastal Health, who decided to waive individual patient consent.

## Author contributions

SD: Data curation, Formal analysis, Writing – original draft, Writing – review & editing. KS: Formal analysis, Methodology, Writing – review & editing. IH: Methodology, Writing – original draft, Writing – review & editing. JL: Formal analysis, Writing – review & editing. JG: Writing – review & editing. NM: Data curation, Methodology, Writing – review & editing. PK: Conceptualization, Formal analysis, Methodology, Writing – original draft, Writing – review & editing.

## Genome Canada Transplant Consortium – members and institutions (only those who provided permission to acknowledge are listed here)

**Canada**: Paul A. Keown, University of British Columbia, Vancouver; Ruth Sapir-Pichhadze, McGill University, Montreal; Stirling Bryan, University of British Columbia, Vancouver; Timothy Caulfield, University of Alberta, Edmonton; Jiannis Ragoussis, McGill University, Montreal; Karim Oualkacha, Université du Quebec à Montreal, Montreal; Kathryn Tinckam, University of Toronto, Toronto; Heloise Cardinal, University of Montreal, Montreal; Sacha A. De Serres, Laval University, Quebec City; Chee Loong Saw, McGill University Health Center, Montreal; Banu Sis, University of Alberta, Edmonton; Karen R. Sherwood, University of British Columbia, Vancouver; Eric Wagner, Laval University, Quebec City; Bruce McManus, University of British Columbia, Vancouver; Robert McMaster, University of British Columbia, Vancouver; Leonard J. Foster, University of British Columbia, Vancouver; Fabio Rossi, University of British Columbia, Vancouver; Christoph Borchers, McGill University, Montreal; Ciriaco A. Piccirillo, McGill University, Montreal; Constantin Polychronakos, McGill University, Montreal; Raymond Ng, University of British Columbia, Vancouver; Anthony Jevnikar, Western University, London; Pieter Cullis, University of British Columbia, Vancouver; Guido Filler, Western University, London; Harvey Wong, University of British Columbia, Vancouver; Bethany Foster, McGill University, Montreal; John Gill, University of British Columbia, Vancouver; Atul Humar, University of Toronto, Toronto; James Lan, University of British Columbia, Vancouver; Prosanto Chaudhury, McGill University Health Centre, Montreal; Bryce Kiberd, Dalhousie University, Halifax; Scott Klarenbach, University of Alberta, Edmonton; Robert Balshaw, University of Manitoba, Winnipeg; Istvan Mucsi, University of Toronto, Toronto; Calvin Stiller, Western University, London; Lynne Senecal, University of Montreal, Montreal; Tom Blydt-Hansen, University of British Columbia, Vancouver. **Germany:** Gerhard Opeiz, University Hospital Heidelberg, Heidelberg; Michael Oellerich, University Medical Center Gottingen, Gottingen. **Netherlands:** Marcel Tilanus, Maastricht University Medical Center, Maastricht; Frans Claas, University of Leiden, Leiden; Teun van Gelder, University of Leiden, Leiden. **United Kingdom:** Steven GE Marsh, Royal Free Campus, London. **United States:** Howard Gebel, Emory University, Atlanta; Eric Weimer, University of North Carolina, Chapel Hill; Bruce Kaplan, Baylor Scott and White Health Systems, Temple.
